# Genome‐scale metabolic models applied for human health and biopharmaceutical engineering

**DOI:** 10.1002/qub2.21

**Published:** 2023-11-13

**Authors:** Feiran Li, Yu Chen, Johan Gustafsson, Hao Wang, Yi Wang, Chong Zhang, Xinhui Xing

**Affiliations:** ^1^ Institute of Biopharmaceutical and Health Engineering Tsinghua Shenzhen International Graduate School Tsinghua University Shenzhen China; ^2^ Key Laboratory of Quantitative Synthetic Biology Shenzhen Institute of Synthetic Biology, Shenzhen Institute of Advanced Technology, Chinese Academy of Sciences Shenzhen China; ^3^ Department of Biology and Biological Engineering Chalmers University of Technology Gothenburg Sweden; ^4^ Key Laboratory for Industrial Biocatalysis Ministry of Education, Institute of Biochemical Engineering, Department of Chemical Engineering Tsinghua University Beijing China; ^5^ Center for Synthetic and Systems Biology Tsinghua University Beijing China; ^6^ Institute of Biomedical Health Technology and Engineering Shenzhen Bay Laboratory Shenzhen China

**Keywords:** constraint‐based modeling, disease, genome‐scale metabolic model, metabolism

## Abstract

Over the last 15 years, genome‐scale metabolic models (GEMs) have been reconstructed for human and model animals, such as mouse and rat, to systematically understand metabolism, simulate multicellular or multi‐tissue interplay, understand human diseases, and guide cell factory design for biopharmaceutical protein production. Here, we describe how metabolic networks can be represented using stoichiometric matrices and well‐defined constraints for flux simulation. Then, we review the history of GEM development for quantitative understanding of *Homo sapiens* and other relevant animals, together with their applications. We describe how model develops from *H*. *sapiens* to other animals and from generic purpose to precise context‐specific simulation. The progress of GEMs for animals greatly expand our systematic understanding of metabolism in human and related animals. We discuss the difficulties and present perspectives on the GEM development and the quest to integrate more biological processes and omics data for future research and translation. We truly hope that this review can inspire new models developed for other mammalian organisms and generate new algorithms for integrating big data to conduct more in‐depth analysis to further make progress on human health and biopharmaceutical engineering.

## 1 INTRODUCTION

Genome‐scale metabolic models (GEMs) describe every known metabolic conversion that can happen in one species and has been a major modeling tool for systematically studying metabolism. Since the first GEM developed in 1999 for *Haemophilus influenzae* Rd [1], GEMs have been reconstructed for more than 6000 organisms till now [2], leveraging the fast development of sequencing techniques and automatic GEM reconstruction tools. Metabolites and reactions annotated from genes are connected through the stoichiometric matrix. By employing quantitative optimization methods such as Flux Balance Analysis (FBA), GEMs facilitate the simulation of metabolic flux distributions, which describe substrate flows from nutrients to all components required for growth. Early attempts in this field include formulating different algorithms to constrain the model to get a reliable flux distribution that could better represent the phenotypes [3, 4, 5]. These developments have led to successful applications of GEMs in guiding metabolic engineering for overproduction of chemicals, fuels, and materials by various microbial cell factories [2, 6].

A better understanding of human metabolism and its relationship with diseases is an important task in human systems biology studies. Meanwhile, animals have been widely used in translational research in recapitulating phenotypic syndromes, clarifying underlying mechanisms, and translating biomedical discoveries toward effective clinical treatments for human disease [7]. Thus, there is an urgent need to develop models for humans and other related animals. However, animals, as complex, multicellular organisms, are more intricate compared to single‐cell microbes. They have specialized cells, tissues, and organ systems that work together to maintain life and perform various functions. Moreover, humans and other animals require a variety of essential nutrients for growth, whereas microbes can use a limited number of substrates to synthesize what they need, making their metabolic processes less complex. Lastly, the genome size of animals is much larger than that of microbes, enabling them to carry out a greater number of metabolic reactions. As a result, GEM development for humans and other animals, as well as the precise internal flux prediction, is much more difficult and demands considerable effort. Recent algorithm development and integration of omics data have facilitated the creation of GEMs for humans and related animals. These developments have improved flux predictions and expanded the applicability of animal GEMs [8]. For example, such predictions can give a systematic and deep understanding of metabolism in a quantitative manner and could potentially revolutionize biopharmaceutical and human health engineering.

Here, we review the genome‐scale modeling of *Homo sapiens* and human health‐related animals, presenting a historical overview of the development of GEMs for various species, represented through a chronological network that shows the inheritance relationships. Databases that are inappropriate for computational analysis, such as HumanCyc for *H. sapiens* [9] and MouseCyc for *Mus musculus* [10], and contain multiple species, such as MetaFishNET for various fish species, are not considered here [11].

## 2 GEM RECONSTRUCTION

**FIGURE 1:**
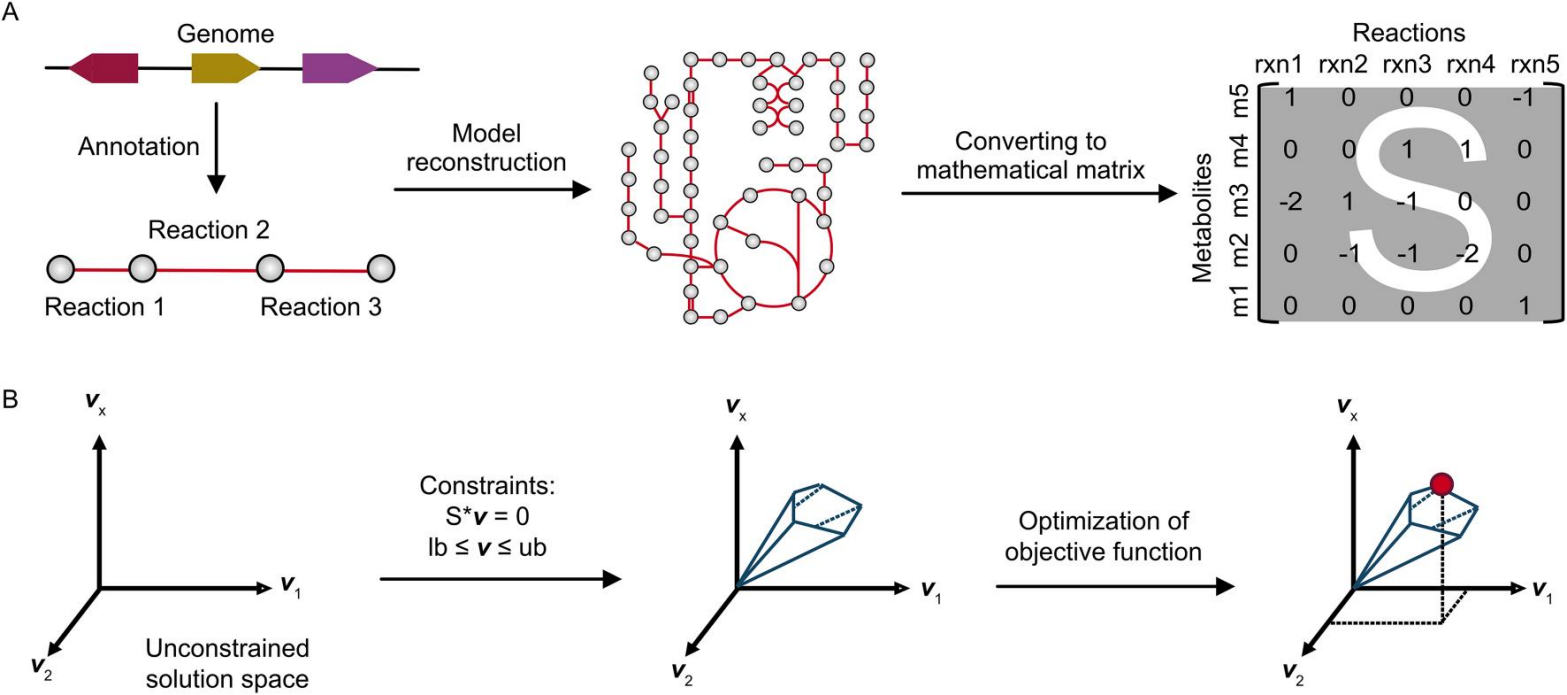
Simplified model reconstruction and simulation process. (A) Model reconsturction process. (B) Model simulation process.

Reconstruction and mathematical modeling processes of GEMs have been extensively reviewed previously [2, 12, 13, 14, 15], and therefore, we only include a brief summary of these concepts here (Figure 1). A prerequisite to the reconstruction of a GEM is the accessibility to whole‐genome sequences. Metabolic genes from a genome are annotated to reactions manually or using automatic pipelines, where reactions are connected via shared metabolites. Then, the reconstructed GEM is converted into a mathematical matrix (S) describing the stoichiometry of the metabolites involved in each reaction and a flux vector (***v***) describing the flux through each reaction. This kind of conversion allows for a quantitative representation of the reaction network. Then, the accumulation rate for each metabolite can be calculated as S × ***v***. However, changes in internal metabolites are difficult to measure at most times. Thus, to simulate the reaction fluxes, researchers impose the steady‐state assumption, that is, internal metabolism is at the steady state in which the metabolite concentration is constant during the simulation (S × ***v*** = 0). Although this assumption is made, the system is still underdetermined, and further constraints are necessary. Typically, lower and upper bounds of reactions must be defined. These bounds for internal reactions usually constrain the flux flow in the thermodynamically favored direction within GEMs. Experimental measurements of exchange rates for nutrient uptake and byproduct production rates can determine reaction bounds for exchange reactions, which serve as additional constraints in the model. Even with these constraints, the problem is usually underdetermined. To address this, FBA assumes that the cell strives to optimize a certain objective function, defined as a linear combination of the fluxes in the model. Then, a set of fluxes can be solved by linear programming with the objective function under the specified constraints. Maximizing growth is extensively utilized in microbial and cancer cell simulations, but objective functions can be different to adapt to the specific scientific context. By integrating different constraints such as transcriptome data and exchange rates for nutrients, the solved different sets of fluxes allow for comparison of metabolic states under given conditions [16]. Therefore, GEM reconstructions and simulations enable quantitative mechanistic investigations of the genotype–phenotype relationship. Thus, a high‐quality GEM reconstruction is critical to enable the systematic understanding of the metabolism of a target organism.

### 2.1 Chronical development of generic GEMs for animals

#### 2.1.1 Homo sapiens

**FIGURE 2:**
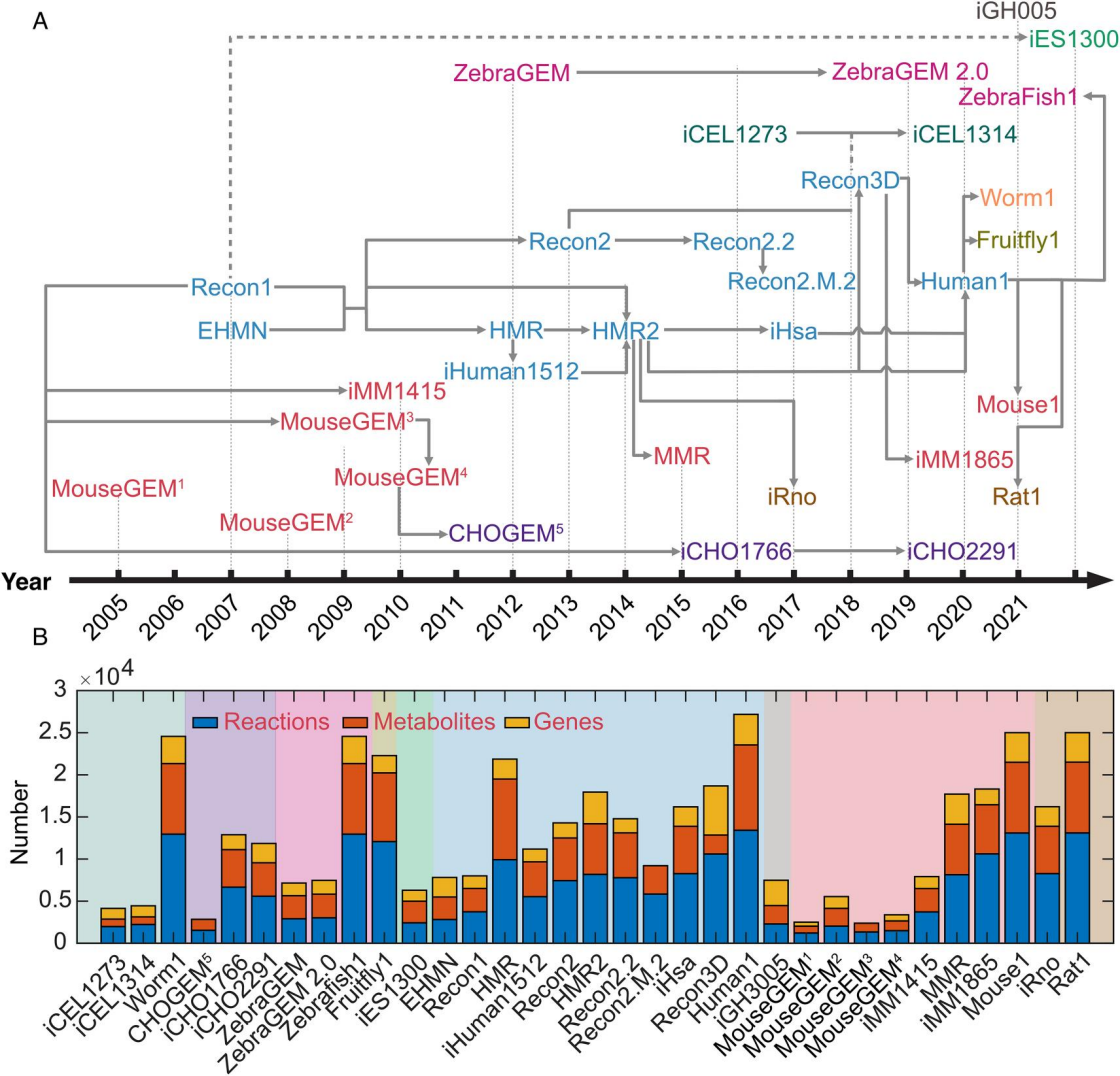
Animal GEM reconstructions. (A) The GEM reconstructed for animals with a historical timeline. (B) The total number of genes, reactions, and metabolites of each GEM. 1, GEM from this reference [18]; 2, GEM from this reference [19]; 3, GEM from this reference [20]; 4, GEM from this reference [21]; 5, GEM from this reference [22].

The first human generic GEM named Recon1 was published in 2007 and reconstructed based on genomic and bibliomic data from 1500 literature sources over 50 years [17] (Figure 2A). This manually reconstructed and literature‐based model, containing eight compartments (cytoplasm, extracellular space, mitochondria, Golgi apparatus, endoplasmic reticulum, lysosome, peroxisome, and nucleus), 3741 reactions, 2766 metabolites, and 1496 genes, is able to perform over 200 known metabolic functions found in a variety of cell and tissue types. Once published, this GEM has been converted into many predictive models used in biomedical applications [23]. In the same year, another GEM for *H. Sapiens*, EHMN, was reconstructed based on multiple database annotations with comprehensive reference ID annotations for reactions and metabolites but did not contain multiple subcellular localizations as in Recon1 [24]. A later release, compartmentalized EHMN, fixed this issue [25].

Following these efforts, multiple GEMs have been developed. The second version of the Recon series, Recon2, published in 2013, combined the effort from the EHMN and several tissue‐specific models for humans [26] (Figure 2A). Due to the tedious work required for a GEM to stay tuned with latest scientific knowledge, modelers of the Recon series decided to adopt a community‐driven approach for GEM updates. Domain experts applied their knowledge to refine and consolidate biochemical knowledge from existing reconstructions and published literature. Recon2 was benchmarked to have improved predictive abilities compared with Recon1. After this, the Recon series went through several impressive updates. Recon2.2 emerged in 2016 with energy production curation and quality control curation for mass and charge balance [27]. Later, Recon2.M.2 arose as an improved version for taking alternative gene splicing into account (both principal and nonprincipal transcripts), serving as a platform to investigate gene–transcript–protein–reaction associations [28]. In 2018, Recon3D included three‐dimensional (3D) information for both metabolites and protein structures; thus, it enables us to assess the sequence variation toward the specific and proteome‐wide effects and perform integrated analysis of human metabolic functions [29]. During the Recon series model update process, there was a significant increase in the number of genes, reactions, and metabolites included in GEMs, representing improved quantitative understanding of gene–protein–reaction associations (GPRs) and human metabolism during these years (Figure 2B).

Another *H. sapiens* GEM series, known as the Human Metabolic Reaction (HMR), has also been continuously updated during these years. The first HMR resulted from merging the Recon1 and EHMN with annotations from databases such as KEGG and HumanCyc [30]. From HMR, the connected model for *H. Sapiens*, iHuman1512, was extracted [30]. The second version of HMR was published in 2014 [31], which was expanded significantly with comprehensive information on fatty acid metabolism. HMR2 was successfully applied to generate tissue‐specific GEMs for studying human hepatocytes and diseases associated with these cells, which will be discussed later in this review. At that time, the HMR series still functioned as a database for extracting information rather than a GEM for performing simulations. Subsequently, HMR2 was expanded by another group giving rise to iHsa with expanding 169 new reactions, 1103 manually reconciled GPRs, and over 5000 additional references to experimental literature and annotation databases [32]. Lastly, the Human1 was published in 2020, which was derived from the HMR series and integrated the Recon lineage, representing the most extensively curated generic *H. sapiens* GEM to date [33]. To enable transparent curation and community contribution, Human1 is hosted on GitHub to record model updates with releases, including scripts, datasets, and the reasoning behind updates, promoting open and parallel collaborations for continuous model development.

Directly analyzing the *H. sapiens* GEM is preferred to unmask the veil of phenotypes, but data from human samples are sometimes difficult to obtain. Animal models and mammalian cells are other essential tools for scientific advancement in providing abundant biomedical data, since they enable experiments that would be unethical to perform on humans. Therefore, to well characterize and understand the metabolism from such experiments, animal GEMs have also been developed to fill this knowledge gap.

#### 2.1.2 Mus musculus

The mouse is recognized as the most important lab animal model in biomedical research for comparative analysis of human gene functions. The experience, knowledge, and advanced techniques developed during a century of research on mice present a unique opportunity to complement and accelerate the exploration of human gene studies through experimental studies of corresponding mouse orthologs. The attempt to reconstruct the GEM for *Mus musculus* can be traced back to 2005, which was based on annotated genomic data and pathway databases [18]. This GEM attempted to account for the carbon, energy, and nitrogen metabolism of the mouse and contained two compartments (cytosol and mitochondria), 1156 gene products, 1220 reactions, and 872 internal metabolites. Even though it was far from complete at the genome scale, considering the low coverage of genes (with only 473 ORFs), it represented the first attempt to collect and characterize the metabolic network for a mammalian organism based on the genome annotation [18]. Then, this model was further updated manually twice in 2009 and 2010, respectively, by integrating additional information on GPRs, improving the stoichiometric balance of reactions in the GEM and filling gaps to improve network connectivity [20, 21]. In parallel, another GEM for *M. musculus* was reconstructed semiautomatically in 2008 based on annotation from databases such as KEGG and MetaCyc, which contain 1399 genes and 2037 reactions compartmentalized to cytosol and mitochondria [19].

Initially, the model reconstruction and curation mainly used a bottom–up approach based on literature search or database annotation [13]. When several well‐curated GEMs became available, researchers started to consider taking benefit of those efforts for GEM reconstruction of other organisms. This was followed by the orthology‐based method, which infers to reconstruct a GEM of a target organism based on previously well‐curated and validated model reconstructions of another reference organism [34, 35]. In 2010, iMM1415 was built using the human Recon1 as the scaffold through homolog searches, given the high sequence homology between mice and humans [36]. This reconstruction contains 1415 genes and 3726 reactions, introducing five additional cellular compartments compared with previously mentioned mice reconstructions [36]. In addition, 260 metabolic tasks were defined to validate the model and served as guidance for the gap filling. Furthermore, this reconstruction took advantage of the availability of gene knockout phenotypes of mouse strains to examine and further improve phenotype prediction capabilities of a mouse GEM. Upon the release of HMR2 for human, the MMR for mouse was constructed using that model as a template, resulting in 8140 reactions, 3579 associated metabolic genes, and 5992 metabolites in eight different compartments [37]. Later, in 2020, with the publication of the more recent human GEM, Recon3D, the mouse GEM was updated to iMM1865 by mapping the human genomic data of Recon3D to mouse [38]. Compared with previously published mouse GEMs, iMM1865 did not contain dead‐end metabolites—metabolites that can only be produced or consumed. Compared with 260 metabolic functions used in previous iMM1415 reconstruction, an expanded set of 431 metabolic functions were utilized during the iMM1865 model validation. The latest mouse genome‐scale metabolic model, Mouse1, was recently published in 2021. This model was constructed using both ortholog‐based mappings of the Human1 [39] and mouse‐specific reactions extracted from KEGG database annotations [39]. Compared with iMM1865, Mouse1 contains more enzyme complexes and scores higher in gene essentiality prediction performance and MEMOTE analysis [40], a benchmark tool for metabolic model performance [39]. Mouse1 was developed in GitHub to facilitate open curation and continuous integration of biochemical knowledge from the research community. Mouse1 is tightly developed with Human1, which promotes the continuous update of the mouse GEM with the human GEM update. Moreover, Mouse1 inherited identical reaction and metabolite IDs from Human1, which is important for translation of results from mice to the human for further analysis of results in the context of human models.

#### 2.1.3 Rattus norvegicus

The rat is another model organism commonly used in preclinical drug development and biomarker discovery. In 2017, the first GEM for *R. norvegicus*, iRno, was reconstructed as an expansion of the HMR2 [32]. Manual curations were performed to ensure rat‐specific metabolic functions, such as de novo vitamin C synthesis and to reduce the redundant isozymes for catalyzing reactions, such as those associated with signaling pathways. In 2021, the latest GEM for rats was published using Human1 as the template. Compared with iRno, Rat1 improves significantly in the number of reactions, metabolites, and genes (Figure 2B) [39].

#### 2.1.4 Cricetulus griseus

Chinese hamster ovary (CHO) cells dominate biotherapeutic protein production and are widely used in mammalian cell line engineering research. To better understand the limitations in protein production and identify potential engineering targets, GEMs for *C. griseus* were created, drawing inspiration from the success of prokaryotic GEMs in cell factory design. The first GEM of *C. griseus* was reconstructed in 2012 using the mouse GEM published in 2010 as the template. This GEM only contained two major compartments, that is, cytosol and mitochondria [22]. Later in 2016, iCHO1766 was developed, which contained 1766 genes and 6663 reactions, describing metabolism and protein production [41]. iCHO1766 was a consensus GEM integrating draft CHO GEM development from different groups using Recon1, Recon2, and Recon2.2 for *H. sapiens* and iMM1415 for *M. musculus* as the scaffold. This GEM performs as a platform for integrating CHO‐relevant big data. Later in 2020, iCHO1766 was updated to iCHO2291, following quality control processes such as curating duplicate metabolite reactions in the model and removing lumped reactions and updating genome annotations [42].

#### 2.1.5 Danio rerio

Fish serve as factories for producing nutrients and compounds with potential health benefits (known as nutraceuticals) that are required by humans. Additionally, they are used as model organisms to study human diseases [43]. To facilitate the understanding of fish metabolism and its use in translational research, GEMs for *D. rerio* have been developed in the past decade. The first GEM for *D. rerio*, ZebraGEM, was published in 2012 using the bottom–up approach by collecting information from KEGG, EntrezGene, Ensembl, fish‐specific database MetaFishNET, and scientific literature [44]. Reactions in this GEM were compartmentalized from manual literature searches. This GEM was validated against known metabolic functions. ZebraGEM did not contain GPRs; thus, it cannot be used to model large screens of single gene knockouts or to integrate gene expression data [45]. To solve this issue, ZebraGEM was updated to 2.0 version, by updating GPRs from the phenotype inconsistency of in silico and in vivo single gene knockouts [45]. Moreover, a functional oxidative phosphorylation pathway was added in this version, which was validated against experimental results. Aforementioned models were based on bottom–up approaches. Until 2021, the Zebrafish GEM integrating the effort from human GEM reconstruction was available as Zebrafish1 [39]. Zebrafish1 expanded substantially, increasing reaction and enzyme complex numbers (Figure 2B).

#### 2.1.6 Litopenaeus vannamei

*L. vannamei*, known as the king shrimp, is the most widely cultured shrimp species. To facilitate the understanding and improve the nutritional quality and yield of shrimp production for human consumption, researchers developed a GEM, iGH3005, for *L. vannamei* in 2021. This GEM was built using the top–down approach of collecting information from databases and literature without adopting any human or fish GEM as the template in the reconstruction process. Then, iGH3005 was used to analyze the difference in nutrient requirements of five commercial varieties for *L. vannamei* and suggested that the feeding for varieties should be adjusted differently [46].

#### 2.1.7 Caenorhabditis elegans

For many years, the Nematoda worm *C. elegans* has served as a model organism for studying human diseases, as well as for investigating relationships between metabolism, nutrition, gene expression, and life history traits. This species has the advantage of being a cost‐effective and genetically tractable model for research. To facilitate metabolic analyses, high‐quality GEMs were also built recently. The first GEM for *C. elegans* iCEL1273 was released in 2016, containing 1273 genes and 1985 metabolic reactions, validated against gene essentiality prediction and other phenotypes [47]. This GEM enabled simulation of the conversion from bacterial biomass into *C. elegans* biomass and the metabolic rewiring in dauer animals against growing larvae. Then, in 2019, this GEM was updated to iCEL1314 including 1314 genes and 2230 reactions by expanding the ascaroside biosynthesis pathway, incorporating transport reactions based on the human Recon3D, manual curations of pseudo‐ and dead‐end genes, and modifications of the composition of sphingolipids [48]. In 2021, the most recent worm GEM, Worm1, was built based on Human1 using the orthology‐based approach, which displayed improved prediction performance compared to iCEL1273 and iCEL1314 [39].

#### 2.1.8 Drosophila melanogaster

*D. melanogaster*, also known as the fruit fly, is one of the well‐studied organisms in biological research, particularly in genetics and developmental biology. It is commonly used to understand genetic control during embryonic development, help develop drugs to combat pathogens, and understand the pathology of neurodegenerative diseases such as Alzheimer’s disease. The GEM for fruit flies, Fruitfly1, was released together with Worm1, Zebrafish1, Mouse1, and Rat1, using the Human1 as the template (Figure 2A) [39].

#### 2.1.9 Gallus gallus

Chicken (*G. gallus*) is the most domesticated species in the poultry industry, and it is also the first sequenced avian creature. The first comprehensive GEM for the chicken was published in 2022, which consists of 2427 reactions, 2569 metabolites, and 1300 genes and was reconstructed manually based on databases including KEGG, BiGG, CHEBI, UNIPROT, REACTOME, and MetaNetX using the bottom–up approach [49].

### 2.2 Context‐specific GEM reconstruction

Human and other animals are multicell and multi‐tissue organisms with complex networks of interactions with well‐differentiated cells, tissues, and organs. Previously mentioned generic GEMs contain all known metabolic reactions that can happen in each organism and is not tissue/cell specific. Therefore, various model extraction algorithms including GIMME [50], iMAT [51], MBA [52], INIT [30], tINIT [53], ftINIT [16] and mCADRE [54], have been developed during the recent decade for generating context‐specific GEMs to investigate specific tissues or cell types. These algorithms can be broadly grouped into two categories: optimization‐based and pruning‐based. Optimization‐based methods use linear or mixed‐integer problem‐solving to generate context‐specific models, with the goal of either removing reactions associated with poorly expressed genes (such as with GIMME) or retaining reactions linked to highly expressed genes (such as with iMAT or INIT). On the other hand, pruning methods identify a set of candidate reactions and then remove them one by one while maximizing the number of reactions removed without impacting the model’s ability to simulate known metabolic processes. There have been comprehensive comparisons and evaluations of these different methods [55, 56, 57]. Generated specific models from these methods have been widely applied in simulating the metabolic phenotypes under particular genetic or environmental perturbations for various applications related to a myriad of biomedical advancements [58].

## 3 APPLICATION OF ANIMAL GEMs

Scientific applications currently conducted on animal‐specific GEMs may be divided into (1) quantitative understanding of metabolic functions, (2) simulating multicellular or multi‐tissue interaction, (3) understanding human diseases, and (4) guiding cell factory design (for a summary, see Table 1).

**TABLE 1:** A selection of model‐based metabolic applications of animal GEMs.

Application	Organisms	Comment	Used reference models	Extraction method	Ref.
Understanding human diseases	*H. sapiens*	COVID‐19	Human1	iMAT, GIMME, INIT, tINIT	[56]
		COVID‐19	Recon3D, Recon1	iMAT, rMAT	[59]
		COVID‐19	Human1	tINIT	[60]
		COVID‐19	Human1	tINIT	[61]
		Hepatocellular carcinoma	HMR2	tINIT	[53]
		Alcoholic hepatitis	Human1	tINIT	[62]
		Non‐alcoholic steatohepatitis	HMR2, HepatoNET1, iLJ1046, iAB676	Consensus model	[31]
		T2D	HMR2	tINIT	[63]
		Inborn errors	Recon2	iMAT	[26]
		Clear cell renal cell carcinoma (ccRCC)	HMR	INIT	[64]
		Neuropsychiatric disorders	Recon3D	iMAT	[65]
		Radiation resistance	Recon3D	Kinetic method	[66]
		Breast cancer	Human1	tINIT	[67]
		Sepsis	Recon1	Fastcore	[68]
Simulating metabolic difference among tissues/cell types		53 healthy tissue metabolic models and 33 cancer metabolic models	Human1	tINIT	[33]
		10 tissues	Recon1	iMAT	[69]
		69 human cell types and 16 cancer types	HMR	INIT	[30]
		65 cell type–specific models	Recon2	iMAT	[26]
		126 human cell types	Recon1	mCADRE	[54]
		Hepatocyte	Recon1	MBA	[52]
		Hepatocyte	HMR2, HepatoNET1, iLJ1046, iAB676	Consensus model	[31]
		Hepatocyte	Recon1, EHMN	Bottom–up approach	[70]
		Hepatocyte	Recon1	GIMME	[71]
		Adipocytes	Recon1	GIMME	[71]
		Adipocytes	HepatoNet1, Recon1, cEHMN	‐	[72]
		Myocytes	HMR2	tINIT	[63]
	*G. morhua*	Hepatocyte	iHepatocytes2322	‐	[73]
	*R. norvegicus*	Hepatocyte	LiverCADRE	GIMME, E‐flux	[74]
Simulating the interaction of multi tissues	*H. sapiens*	Multi‐tissue	Recon1	MBA	[75]
		Multi‐tissue	Recon1	GIMME	[71]
		Multi‐tissue, whole‐body	Recon3D	Fastcore	[76]
Cell factory design	CHO	Protein production	CHO‐S model	‐	[77]
		IgG production	CHO‐K1 model	‐	[78]

### 3.1 Understanding metabolic functions

Based upon the generic human metabolic network, Recon1, context‐specific models were reconstructed for 10 different human tissues in 2008 using iMAT [69] and for around 100 human tissues using mCADRE in 2012 [54]. Another generic human GEM HMR was used to generate specific models for 69 human cell types using the INIT method [30]. Later, Recon2 was used to generate specific models for 65 cell types [26]. Human1 was used to generate specific models for 53 healthy tissues and 33 cancer cell types. Among all tissues, hepatocytes, adipocytes, and astrocytes gained more attention with manual curation and multiple updates. As for human hepatocytes, there are HepatoNet1 [70], iLJ1046 [52], iAB676 [71], liverCADRE [54], iHepatocyte1154 [30] and iHepatocytes2322 [31]. Among them, iHepatocytes2322 is a consensus model which integrates previous models for human hepatocytes. There are iAB586 [71] and iAdipocytes1809 [72] for human adipocytes and iCM3765 [79] for human astrocytes. All those GEMs have been used to characterize specific metabolic functions of cell types/tissues.

Besides *H. sapiens*, there are multiple tissue‐specific models for other animals, such as ReCodLiver0.9 [73], which was built for the liver of Atlantic cod using iHepatocytes2322 as the template and iRatLiver, which was built for rat liver using liverCADRE as the template [80]. Both models were used to predict metabolic changes under context conditions. As for CHO cells, cell‐line‐specific models for CHO‐K1, ‐S, and ‐DG44 were constructed using the GIMME algorithm for metabolic simulation, which provided the biochemical basis of growth and recombinant protein production [41].

### 3.2 Understanding human diseases

While context‐specific GEMs can help in elucidating phenotypic patterns among tissues, their practical value lies in their ability to aid in the identification of biomarkers and drug targets for the development of novel diagnostic methods and treatments.

The utilization of GEMs to understand and facilitate cancer research has gained more interests during these years. Generic cancer metabolic models were firstly constructed to simulate the common metabolic attributes of cancers, such as ATP production, growth, and the Warburg effect, that is, the conversion of glucose to lactate in the presence of oxygen and functioning mitochondria. There are several generic cancer models that are focused on simulating the metabolic rewiring of cancers [81, 82]. Later, GEMs were built for specific cancers to model the heterogeneity among cancers, such as for 16 cancer types [30] to identify cancer reporter metabolites and for 33 cancer types to simulate metabolic differences compared with healthy tissues for identification of possible treatment [33]. On the other hand, there might be significant changes in metabolism for the same cancer cells surviving in its changing microenvironment during tumorigenesis [83] and metastasis [84]. Recently, GEMs were also used to identify metabolic adaptions of metastatic triple negative breast cancer with its microenvironment [67]. Besides identification of cancer‐specific metabolic features, there is an ultimate desire to utilize the gained knowledge to guide cancer treatment. Multiple studies integrated clinical data with GEMs to facilitate the analysis, such as integrating high‐quality personalized proteome data for hepatocellular carcinoma patients with HMR2 in order to facilitate the identification of anticancer drugs for hepatocellular carcinoma [53], discovering metabolite biomarkers for nonalcoholic steatohepatitis [31], predicting potential therapeutic interventions for alcoholic hepatitis [62], identifying potential targets for clear cell renal cell carcinoma [64], predicting therapeutic metabolic gene knockouts for neuropsychiatric disorders [65], identifying biomarkers for classifying type 2 diabetes (T2D) samples [63] and predicting changes in metabolite biomarkers for 49 inborn errors of metabolism [26].

GEMs can also predict altered intracellular metabolic states caused by acute diseases such as viral infections [59, 60] and sepsis [68]. During this COVID‐19 pandemic, human GEMs have been used to systematically understand and address this complex disease and dynamics combined with various datasets and analysis methods. Gene expression datasets of infected and normal human bronchial epithelial cells were integrated with Human1 using tINIT. In this study, lipid metabolism was identified as the most affected pathway, which confirms clinical metabolomics studies [60]. Multiple model extraction methods such as iMAT, GIMME, INIT, and tINIT were applied to Human1 with transcriptomes of healthy and COVID‐19‐specific various cell lines/tissues [56]. They indicated that models extracted from GIMME and tINIT models provided the most biologically relevant results and should have a larger emphasis on further analyses. Moreover, models from tINIT in this study have predicted the lack of vitamins in COVID‐19 patients. In another study, Human1 was integrated with transcriptome data using tINIT and suggested that metabolic perturbations of the TCA cycle could be a treatment for severe COVID‐19, which was further supported by sctMetabolomics of monocytes [61]. Other human GEMs, Recon3D and Recon1, were also used to integrate various human gene expression datasets from COVID‐19 infected and noninfected controls. The study identified anti‐SARS‐CoV‐2 targets that counteract the metabolic changes induced by the virus and experimentally validated these targets using siRNA assays [59].

### 3.3 Simulating the interaction of multiple cell types/tissues

As mentioned, humans are multicellular organisms with complex networks of interactions. Genome‐scale modeling efforts have been utilized to simulate and analyze these complex biological systems. One initial genome‐scale attempt was to model the interaction of three specific tissues of hepatocytes, adipocytes, and myocytes [71], which was applied to simulate known integrated metabolic cycles and to determine metabolic variations between obese and type II obese gastric bypass patients in the integrated multi‐tissue context. Another genome‐scale multi‐tissue model was reconstructed to study T2D in MKR mice, containing specific models of adipocytes, hepatocytes, and skeletal muscle tissue derived from Recon1. The downregulation of branched‐chain amino acid and fatty acid oxidation in MKR mice was identified [75]. Compared with previously mentioned efforts of the three tissues, the first sex‐specific whole‐body GEMs were reconstructed in 2020, each containing 26 organs and six blood cell types [76]. These models were parameterized with physiological, dietary, and metabolic data, which were validated by predicting known biomarkers for inherited diseases [76] and used to analyze diseases such as type 1 diabetes [85], COVID‐19 [86] and inborn errors of metabolism [87].

### 3.4 Cell factory design

GEMs have long been used to guide cell factory designs by providing effective gene manipulations for the enhanced production of chemicals in microorganisms [2, 6, 88]. For mammalian therapeutic protein production, CHO cells serve as the most prevalent hosts producing over 70% share of monoclonal antibodies on the market [89, 90]. Even though the production titer of the CHO has significantly improved during the last decade, it is still far from the maximal theoretical yield. Thus, there is rising interest in using GEMs to understand CHO metabolism systematically and further identify engineering targets for protein overproduction. In this regard, reconstructed cell line‐specific models for CHO‐K1, ‐S, and ‐DG44 from iCHO1766 were used to evaluate bioprocessing strategies and metabolic engineering approaches toward protein overproduction, suggesting that cell engineering could enhance the efficiency of resource utilization compared to common bioprocess treatments [41]. Genome‐scale CHO models have also been applied to select preferred CHO clones with stable genomes and high productivity [91], optimize culture media for IgG production [78], and identify metabolic bottlenecks [77]. Since protein production involves the complex protein modification process besides the metabolism, a model integrated the GEM with protein secretory pathway add‐ons; these were developed to understand protein secretory capacities and identify the bottleneck in protein production [92].

## 4 CHALLENGES AND FUTURE PERSPECTIVES

Genome‐scale modeling of animals has been evolving for more than 15 years, contributing to gaining insights into metabolic processes and applications toward human health and benefits. However, challenges still need to be addressed in the future.

### 4.1 High‐quality generic GEM development and curation

Animal metabolism is more complex, and the scale of metabolic reactions is larger than that of microbes, making the development of animal GEMs much more difficult. Thus, compared to the mature microbial GEM development, the animal GEM reconstruction is still at its fast‐developing stage. These challenges include knowledge gaps in uncharacterized enzymes, lack of definition of objective functions, and lack of a straightforward evaluation method of model quality. The current human GEM and derived animal GEM reconstructions inherited many GPR annotations from the first several GEMs, such as EHMN and Recon1, in which ambiguous annotations may have been updated and therefore remain to be curated (Figure 2A). The reaction coverage of human GEMs has tremendously increased during recent development. However, even for the latest GEM, Human1, many dead‐end metabolites remain. These dead‐end metabolites indicate knowledge gaps that could be filled by adding new reactions supported by experimental evidence. Currently, the vast underground metabolism is not considered in the canonical GEM reconstruction [93]. In the future, as more detailed phenotypic experiment data are gathered and new algorithms are developed, researchers may be able to reconstruct a more comprehensive GEM. Another question is the selection of objective function in model simulations. As one of the most important parameters in the GEM, objective function representing the cell behavior needs to be chosen for accurate flux predictions. The regime of the objective function for maximizing growth in the cases of microbes seems intuitive and useful. However, growth maximization is inappropriate for animal cells, tissues, organs, and especially the healthy body, which barely grows. There are several alternatives, such as maximization of weighted combination of ATP production and biomass [94], efficient use of enzyme capacity [66], maximization of energy production [95], minimization of enzyme cost [33, 42], and maximization of the Pearson correlation between a flux vector and its corresponding gene expression data [62]. The evaluation of objective functions should be sufficiently considered during the simulation of animal GEMs. Moreover, considering the much more complex media or diet compositions for animals than microbes, the flux distribution can be significantly diverse with different parameterizations in animal GEMs. Therefore, sufficient data for metabolite exchange rates must be considered while constraining the model. Another improvement could be the model evaluation part, which currently primarily relies on binary tests, based on for example, metabolic tasks and gene essentiality for cell line‐specific models. Higher availability of fluxomics data could provide additional means to evaluate model performance.

### 4.2 Integrating biological processes

Human diseases are highly complex, involving massive signal cascading. Thus, GEM‐based simulations alone are sometimes not enough to identify causes and mechanisms for complex diseases. Therefore, in the future, more biological processes must be integrated besides metabolism. There are several attempts, such as the integration of regulatory networks [85, 96], protein secretory pathways [92], and enzyme constraints [33, 42] into the GEM to further improve the model predictive power. One of the most significant developments has been the formulation and implementation of protein synthesis with the metabolism to computationally predict individual gene expression, also known as the metabolism and expression model [97] or fine‐grained proteome‐constrained model [88], which can be done de novo without the need for experimental phenotype data. This ability to computationally simulate the cellular function can lead to understanding the mechanisms for gene expressions among tissues and diseased states. Recent advances in integrating cofactor usage into GEMs have successfully been utilized to simulate iron deficiency for yeast [98]. This iron deficiency is a common complication for cancer patients, especially those with solid tumors [99]. By incorporating this approach, researchers can model the metabolic changes that occur during iron deficiency, which is a common complication for cancer patients and can contribute to the development of solid tumors. We believe that it will ultimately be necessary to integrate many other biological processes to fully integrate more information flow with the metabolism to give accurate internal flux predictions and insightful guidance about human disease.

### 4.3 Integrating omics data

GEM development relies heavily on technological advances. With technological advances in profiling metabolomes, the metabolite scope of the GEM can be defined, and knowledge gaps can be deducted and guided for further experimental design to characterize enzyme functions [93]. Advances in measurements of transcriptome and proteome data for more tissues/cells and accounting for the splicing variant information would help to reconstruct high‐quality models, which is especially important when gene expression and protein synthesis are considered in the model scope in the future. Moreover, the improvement in single‐cell RNA sequencing [16] and spatial transcriptomes [100, 101] would be valuable for reconstructing the specific model to analyze the metabolic interplay between different cell types in organs. Lastly, fluxomics data would be valuable to shrink the solution space and serve as important data for model evaluation.

### 4.4 Whole‐body simulation and personalized medicine

In order to describe the overall human metabolism, whole‐body models were utilized to simulate whole‐body functions and predict each tissue’s contribution toward the total body phenotype [76]. However, modeling multi‐tissue interaction still presents many technical issues. For example, differentiated functions in multiple cell types and subcompartments in complex organs are not represented in current whole‐body models, which may be improved by the development and incorporation of single‐cell and spatial omics data. Moreover, with the development of mature human organoids [102], measured data can be used to infer missing parameters and constraints, which can also be used to validate model reconstructions. As for personalized medicine perspective, we believe that model predictions can be significantly improved if more data can be gathered from the same patient, such as the metabolome of body fluids, dietary habits, lifestyle, and physical activities. With such data being integrated into the more advanced whole‐body model, the digital twin of human and personalized precision medicine can truly emerge.

## AUTHOR CONTRIBUTIONS

**Feiran Li**: Conceptualization and writing. **Yu Chen**: Conceptualization; writing – review and editing. **Johan Gustafsson**: Writing – review and editing. **Hao Wang**: Writing – review and editing. **Yi Wang**: Writing – review and editing. **Chong Zhang**: Writing – review and editing. **Xinhui Xing**: Supervision; Writing – review and editing.

## CONFLICT OF INTEREST STATEMENT

The authors Feiran Li, Yu Chen, Johan Gustafsson, Hao Wang, Yi Wang, Chong Zhang, and Xinhui Xing declare that they have no conflicts of interests.

## ETHICS STATEMENT

This article is a review article and does not contain any studies with human or animal subjects performed by any of the authors.
